# Temporal Development of Gut Microbiota in Triclocarban Exposed Pregnant and Neonatal Rats

**DOI:** 10.1038/srep33430

**Published:** 2016-09-20

**Authors:** Rebekah C. Kennedy, Russell R. Fling, Michael S. Robeson, Arnold M. Saxton, Robert L. Donnell, John L. Darcy, David A. Bemis, Jiang Liu, Ling Zhao, Jiangang Chen

**Affiliations:** 1 Department of Public Health, The University of Tennessee, Knoxville, TN 37996, USA; 2 Comparative and Experimental Medicine, The University of Tennessee, Knoxville, TN 37996, USA; 3 Department of Microbiology, The University of Tennessee, Knoxville, TN 37996, USA; 4 Department of Fish, Wildlife & Conservation Biology, Colorado State University, Fort Collins, CO 80523, USA; 5 USDA APHIS, National Wildlife Research Center, Fort Collins, CO 80521, USA; 6 Department of Animal Science, The University of Tennessee, Knoxville, TN 37996, USA; 7 Department of Biomedical and Diagnostic Sciences, The University of Tennessee, Knoxville, TN 37996, USA; 8 Department of Ecology and Evolutionary Biology, University of Colorado, Boulder, CO 80309, USA; 9 Department of Nutrition, The University of Tennessee Knoxville, TN 37996, USA.

## Abstract

Alteration of gut microbial colonization process may influence susceptibility of the newborn/infant to infectious and chronic disease. Infectious disease risk leads to widespread use of non-prescription antimicrobials in household products such as Triclocarban (TCC), an antimicrobial compound in personal care products. TCC concentrates in and is transferred through the milk to suckling offspring. TCC exposure during gestation and lactation significantly reduced phylogenetic diversity (PD) among exposed dams and neonates. Among dams using weighted UniFrac distances, TCC induced significant dysbiosis of gut microbiota by gestational day (GD) 18, a trend that continued after delivery. Similarly, an overall restructuring of gut microbiota occurred in neonates. By postnatal day (PND) 12, communities separated based on exposure status and became significantly different at PND 16. The ability of TCC to drive microbial dysbiosis warrants future investigation to evaluate the safety of non-prescription antimicrobial use, including TCC, during critical exposure windows.

Though it has commonly been estimated that bacteria cells outnumber human cells 10 to 1, more recent estimates suggest that a 1 to 1 ratio may be a more appropriate calculation^1^. The collective gut microbiota can act as an “ancillary organ” with a critical function in human health including regulation of host metabolism and energy balance, immune function stimulation, maintenance of host nutritional physiological homeostasis, and defense against pathogens^2^,^3^. Indigenous gut microorganisms occupy available intestinal niches, therefore any transient species derived from the environment will not colonize and will instead pass through the gastrointestinal system^4^. In contrast, alteration or imbalance of the composition of commensal bacterial population could induce transient or permanent damage to the host with profound health consequences^5^.

At birth, microbial colonization is largely a product of the host environment and is tied to a variety of factors including delivery (vaginal/caeseran) and infant feeding mode (breast/formula feeding)^6^,^7^. The evolvement of infant gut microbial composition is dynamic with large community shifts that occur at transitional stages, i.e. when solid foods are introduced or during early exposure to prescription antibiotics^8^,^9^. Close to the conclusion of the first year, the infant acquires a less dynamic gut microbial community that gradually converges to a more adult-like profile^8^.

The composition of the gut microflora can have a broad impact on the health of the host; it is well established that prescription antibiotic exposure can disrupt the balance of the intestinal microbiota potentially leading to unintentional side-effects; alteration of the colonization process may influence susceptibility of newborn/infant to infectious disease in the short-term and lead to immune mediated and metabolic disorders later in life^3^,^5^. More than 40% of pregnant women are prophylactically prescribed antibiotics during pregnancy/birth for prevention of preterm labor, intrapartum fever, vertical pathogen transmission to the infant, and maternal morbidity after caesarean section^10^. While the average pregnant or lactating female does not control the use of prescription antibiotics, precaution and fear of infectious disease outbreak in human populations leads to widespread use of non-prescription antimicrobials in household products^11^,^12^. It is estimated that more than 10 million women are either pregnant or lactating in the United States at any given time and the use of antimicrobial personal care products occurs among this population without confidence in the safety of antimicrobial exposure during early-life periods^11^,^12^,^13^. Specifically, the impact of early-life non-prescription antimicrobial compound exposure on both intestinal microbiota community integrity and the resultant health outcomes are understudied.

Triclocarban (3,4,4′-trichlorocarbanilide; TCC) is a high production volume antimicrobial, used in personal care products, at a mass of up to 1.5% in certain brands of bar soaps^11^,^14^. TCC can be absorbed through the skin and has been detected in adult human urine, serum and in cord blood indicating exposure throughout the lifespan^15^,^16^,^17^. Volunteers with frequent exposure through the application of TCC containing products tend to have a higher TCC body burden in the circulation^18^. Recent evidence demonstrates that human exposure to TCC may not be limited to the purposeful use of antimicrobial products, but could occur through the diet^19^. Following incomplete removal by wastewater treatment process, TCC is detected in nutrient-rich sludge that may be applied as agricultural fertilizer leading to safety concerns regarding the potential intake through the food chain^20^.

Previously our group reported that TCC concentrates in the breast milk after dietary exposure in a rodent model indicating potential neonatal TCC exposure via lactation^21^. Furthermore, neonates with TCC exposure during lactation had distended gastrointestinal tracts with liquid, mustard-colored diarrhea implying the disturbance of intestinal microbiota and hence a dysbiotic status. In this report, we utilized a rodent model to investigate and characterize the temporal dynamics of intestinal microbiota in pregnant dams and neonatal rats in response to TCC exposure during gestation and lactation.

## Results

### Influence of Gestational and Lactational TCC Exposure on Dam Fecal Microbiota

#### Alpha and Beta Diversity

**Figure 1:**
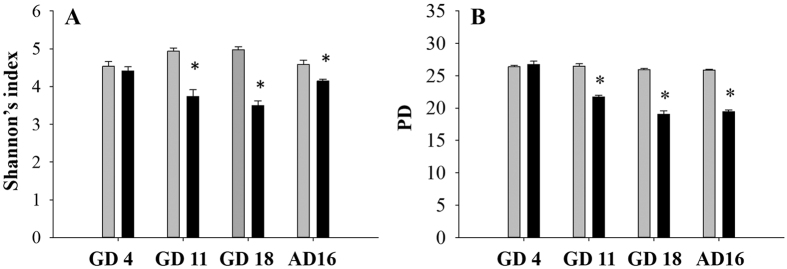
Alpha diversity of dams during gestation and lactation. Shannon’s diversity index (**A**) and phylogenetic diversity (**B**) is shown at Gestational day (GD) 4 (baseline), 11, 18 and 16 days after delivery (AD) (control: gray bar, 0.1% w/w TCC: black bar; n = 4/group). Data represent mean ± SEM of each group. Data were analyzed with two-way ANOVA with repeated measures followed by Bonferroni post-hoc test. Statistical significance was set at p = 0.05; (*) indicates statistical significance at each time point relative to controls.

After quality filtering and removal of any OTU present at less than 0.005% of the total read count, 54 samples comprised of 4,931,803 sequences remained with an average of 91,330 sequences per sample. Neither Shannon’s index nor phylogenetic diversity differed between control and exposed dams prior to TCC exposure at baseline (GD 4). TCC exposure significantly reduced the diversity of microbiota in feces of treated animals compared to controls at 7 days after treatment (GD 11: Shannon 4.93 ± 0.088 vs 3.73 ± 0.180, Fig. 1A; PD 26.5 ± 0.338 vs 21.7 ± 0.277, Fig. 1B). This trend continued throughout gestation after 14 days of treatment at GD 18 (Shannon 4.97 ± 0.077 vs 3.50 ± 0.123, Fig. 1A; PD 25.9 ± 0.204 vs 19.1 ± 0.522, Fig. 1B) and into lactation 16 days after delivery (AD 16), corresponding to 34 days of TCC exposure when both measures were significantly suppressed in the exposed dams compared to controls (Shannon 4.59 ± 0.109 vs 4.14 ± 0.051, Fig. 1A; PD 25.9 ± 0.143 vs 19.4 ± 0.272, Fig. 1B; two-way ANOVA, p < 0.05).

**Figure 2:**
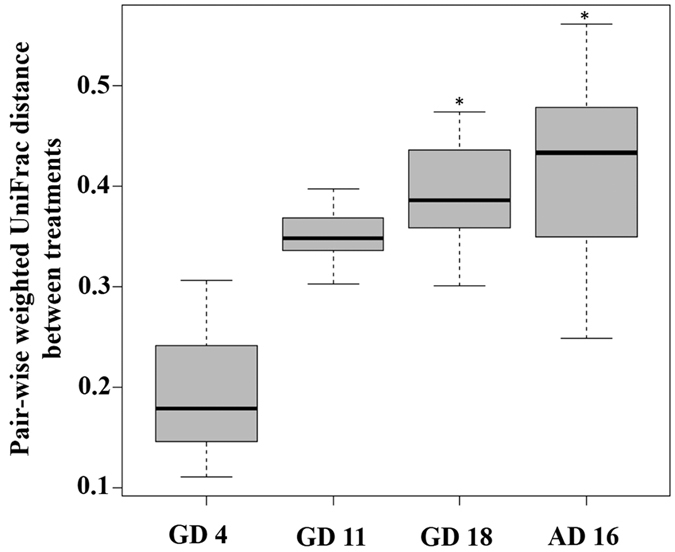
Beta diversity of dams during gestation and lactation. Pairwise weighted UniFrac distance between TCC exposed and control dams is shown among dams at Gestational Days (GD) 4 (baseline), 11, 18 and 16 days after delivery (AD); n = 4/group. Statistical significance of community level microbial distance was analyzed with Adonis, in the Vegan package, at each collection date^50^,^51^. Repeated measures analysis was conducted and significant time-treatment interactions were investigated with the Vegan package. (*) indicates statistical significance at each time point relative to controls.

Increasing microbial community dissimilarity of TCC exposed vs control dams was pronounced at GD11 and maintained over time (Fig. 2, Supplementary Fig. S1). Microbiota structure became statistically dissimilar by 14 days of treatment (GD 18; R^2^ = 0.69, Adonis p < 0.05) and remained different until AD 16 (34 days of TCC exposure; R^2^ = 0.69, Adonis p < 0.05). Repeated measures analysis revealed a significant interaction between time and treatment (R^2^ = 0.27, Adonis p < 0.05). Post-hoc analysis demonstrated that a significant time-treatment interaction occurred beginning at GD 11 after which microbial communities in control and treatment dams behaved differently.

#### Fecal Microbiota Community Composition

**Figure 3:**
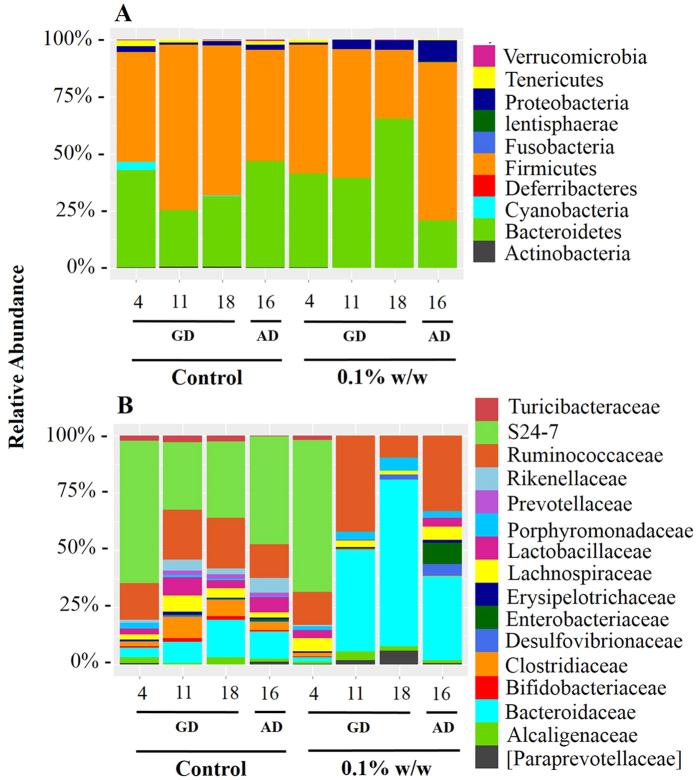
Relative abundance of bacteria among dams at the phylum (**A**) and family (**B**) levels by collection date at GDs 4 (baseline), 11, 18 and 16 days after delivery (AD) (n = 4/group). At the family level, only the top 100 OTUs are shown. Taxon labeled within square brackets indicate GreenGenes proposed taxonomy.

Figure 3 shows the relative abundance of the gut microbial community composition of dams over time, during pregnancy and lactation. Visually, the effect of TCC was more pronounced at the family than the phylum level (Fig. 3A,B). Across the study period in control dams, *S24-7* dominated fecal microbiota. While the *Bacteroideaceae* family was overrepresented in the microbiota of dams exposed to 0.1% w/w TCC. Significant differences at the phylum and family level across time and exposure groups are shown in Tables 1 and 2 respectively.

### Influence of Gestational and Lactational TCC Exposure on Neonatal Microbiota

#### Alpha and Beta Diversity

**Figure 4:**
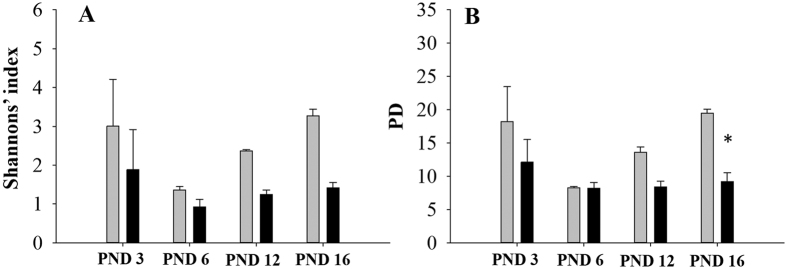
Alpha diversity of neonates during lactation period. Shannon’s diversity index (**A**) and phylogenetic diversity (**B**) is shown at postnatal days (PND) 3, 6, 12 and 16 (control: gray bar, 0.1% w/w TCC: black bar; n = 3/group). Data represent mean ± SEM of each group. ANCOVA at each individual time point was conducted using alpha diversity of dams at GD 18 as the covariate for phylogenetic diversity and Shannon’s index followed by Bonferroni post hoc test. Statistical significance was set at p = 0.05; (*) indicates statistical significance at each time point relative to controls.

**Figure 5:**
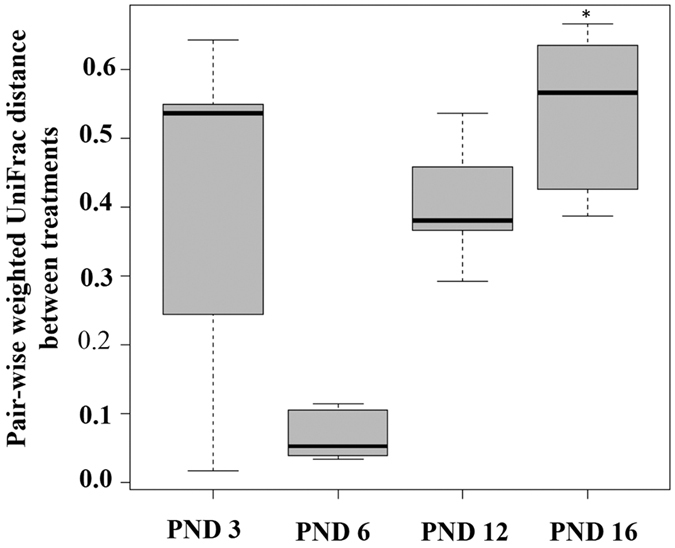
Beta diversity of neonates during lactation period. Pairwise weighted UniFrac distance between TCC exposed and control neonates is shown at postnatal days (PND) 3, 6, 12 and 16 (n = 3/group). Community level statistical significance was analyzed using Adonis, in the Vegan package^50^,^51^. Repeated measures analysis was conducted and significant time-treatment interactions were investigated with the Vegan package. (*) indicates statistical significance at each time point relative to controls.

Among neonates, Shannon’s index did not differ between the two groups across the study period Fig. 4A. Phylogenetic diversity became significantly different on PND 16 (PD 19.51 ± 0.59 vs 9.18 ± 1.35 Fig. 4B; ANCOVA, p < 0.05). The effect of TCC exposure on beta diversity is shown in Fig. 5 and Supplementary Fig. S2 using weighted UniFrac distances. Regardless of treatment status, an initial stochastic pattern emerged at PND 3 followed by convergence at PND 6. After PND 6, the weighted UniFrac distances behaved similarly between control and exposed groups. By PND 12, separation based on treatment status occurred which became significantly different at PND 16 (R^2^ = 0.87, Adonis p < 0.05). Repeated measures analysis revealed an effect of collection date (R^2^ = 0.25).

**Figure 6:**
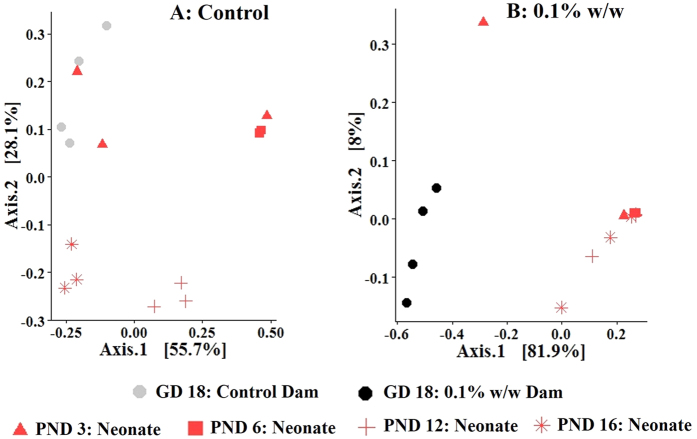
Comparison of the principal coordinate analysis of weighted UniFrac distances among samples collected from (**A**) control dams (gray circles, n = 4) at GD 18 and samples collected from their offspring (n = 3) at PNDs 3 (triangle), 6 (square), 12 (cross) and 16 (star); (**B**): 0.1% w/w TCC exposed dams (black circles, n = 4) at GD 18 and samples collected from their offspring (n = 3) at PNDs 3 (triangle), 6 (square), 12 (cross) and 16 (star).

Given the pattern of diversity shown in Fig. 5 and Supplementary Fig. S2, where a variable configuration arises at PND 3, followed by reorganization and subsequent separation based on exposure status coupled with the common assumption that microbiota is transferred early on from maternal sources to neonates, we sought to understand microbial similarity between dam samples collected near birth and neonate samples collected throughout the study period. To do so, we further compared weighted UniFrac distances of neonatal samples during lactation in relation to dam samples at GD 18. Figure 6A demonstrates initial clustering between control dams at GD 18 and control neonates at PND 3; neonatal diversity then reorganized, moving away from dams at PND 6 and again clustered more closely with dams at PND 12 and PND 16. The visualization between TCC exposed dams and exposed neonates is shown in Fig. 6B. Note that samples of exposed neonates were isolated away from dams at all time points.

#### Cecum Microbiota Community Composition

**Figure 7:**
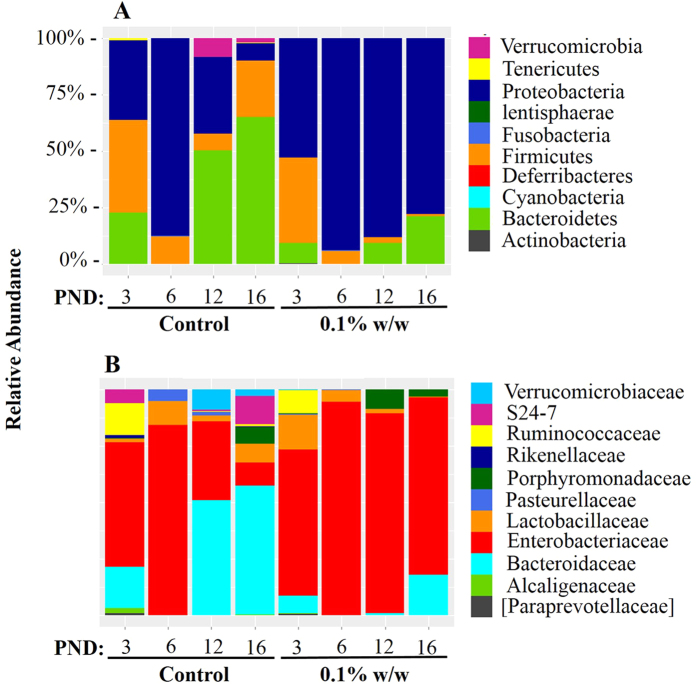
Relative abundance of bacteria among neonates at the phylum (**A**) and family (**B**) levels by collection date at PNDs 3, 6, 12 and 16 (n = 3/group). At the family level, only the top 50 OTUs are shown. Taxon labels within square brackets indicate GreenGenes proposed taxonomy.

Relative abundance of microbiota present in neonate samples revealed a dominance by three phyla: *Proteobacteria*, *Firmicutes*, and *Bacteroidetes* regardless of collection date or treatment status (Fig. 7A). While increases in the proportion of *Bacteroidaceae* are noted in microbiota collected from control samples, *Enterobacteriaceae* dominated samples collected from neonates suckled by 0.1% w/wTCC exposed dams (Fig. 7B). Significant differences at the phylum and family level across time and exposure groups are shown in Table 3 and Table 4 respectively.

### TCC Exposure on Pathology of Neonatal Gastrointestinal Tract

**Figure 8:**
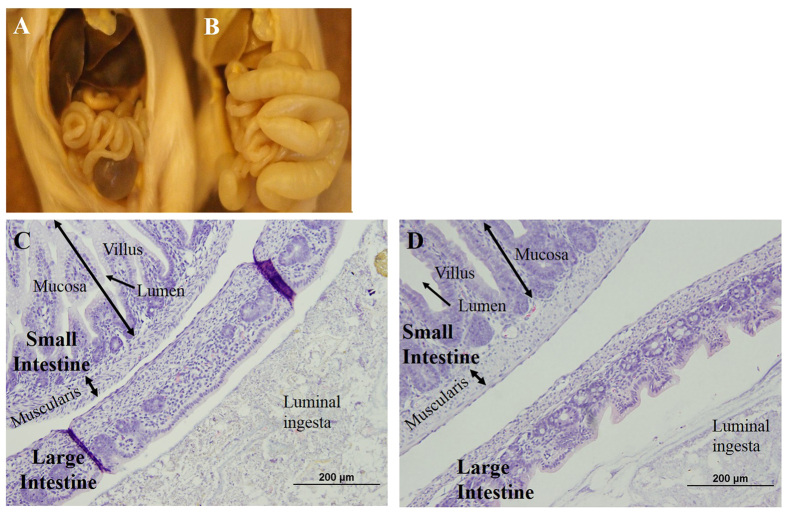
Representative histology of formalin fixed gross gastrointestinal morphology in control (**A**) and 0.1% w/w TCC (**B**) exposed male neonates at PND 12. Large intestine (cecum) and small intestine (jejunum) is shown from control (**C**) and 0.1% w/w TCC (**D**) exposed neonates at PND 12 (H&E, 20X).

Grossly, TCC exposure during gestation and lactation led to enlarged abdomens with mustard colored diarrhea in neonates. The formalin fixed gut of a control and an exposed neonate at PND 12 are shown in Fig. 8A,B respectively. Compared to controls, the gastrointestinal tracts of TCC exposed neonates were filled with gas and liquid. H&E staining of the large (cecum) and small intestine (jejunum) is shown between control (Fig. 8C) and TCC exposed neonates (Fig. 8D) at PND 12. No apparent histological differences were noted between the two groups.

## Discussion

It is common to choose non-prescription antimicrobial containing products for prophylactic reasons^11^,^12^. TCC has been detected in human serum and cord blood suggesting a systemic distribution of this hydrophobic compound through maternal circulation^16^,^17^. Hydrophobic drugs are likely to concentrate in breast milk because of the high lipid load^22^. Detection of TCC is reported in human milk, implying that as the natural and optimal food for infants, breast milk may serve as the primary exposure route to TCC among breastfed infants^23^. We recently demonstrated that TCC was transferred through the milk to suckling neonates^21^. Pups exposed to TCC through lactation had distended gastrointestinal tracts with liquid, mustard-colored diarrhea. Further, the concentration of TCC identified in the milk of exposed dams was four times higher than the corresponding levels found in maternal circulation^21^. The potential TCC exposure among nursing infants dictates the need to investigate the effect of TCC on the gut microbiota composition during early life.

In this study, to understand the development of gut microbial community structure in neonatal rats and understand the dynamics of pregnant/lactating rats in response to TCC exposure, fecal/cecum material were collected at specific time-points during gestation and lactation. Provision of TCC during gestation and lactation altered the community structure of dam fecal microbiota over time. In dams, alpha diversity was significantly reduced in exposed animals at all collection dates after baseline (Fig. 1A,B). Beta diversity was significantly dissimilar on both GD 18 during gestation and 16 days post-delivery (AD 16) in exposed compared to control dams (Fig. 2, Supplementary Fig. S1). Weighted UniFrac Adonis stratified to each sample over time revealed a significant interaction between time and treatment that occurred at GD 11. Here, distances remain relatively stable among exposed dams across the study while microbial distance among control animals becomes more dynamic after GD 11 (Supplementary Fig. S1). Thus, it appears that TCC as an antimicrobial confines distance of exposed dams relative to controls.

In the dam, the effect of TCC exposure on beta and alpha diversity was mirrored in the microbial relative abundance at the phylum and family level (Fig. 3A,B). As shown in Table 1 and Table 2, the majority of microbiota that increased in relative abundance following exposure to 0.1% w/w TCC, stain Gram-negative. In addition, *Clostridiaceae*, *Lactobacillaceae* and *Turicibacteraceae* that stain Gram-positive, made up a significantly increased proportion of the relative abundance in control samples compared to TCC exposed samples. This is not altogether surprising given that TCC shows primary efficacy for Gram-positive bacteria^24^. This selectivity could allow for the overgrowth of potentially pathogenic Gram-negative strains. Though overt signs of infection were not observed in the dam. Because we did not observe adverse gastrointestinal reactions (i.e. diarrhea) in the adult animals during the study period, histological assessment was not conducted.

During early life, one view of antibiotic-induced dysbiosis purports that the gut microbiome responds to prescription antibiotics with the loss of keystone taxa and metabolic shifts in the short-term^5^. Even after antibiotic treatment ends, keystone taxa may not have recovered and the loss of diversity could allow for the bloom of pathogens and pathobionts. In this study, unexposed neonatal communities became more diverse over time while TCC exposure, like prescription antibiotics^5^, restricted diversity of colonizing species during the same period (Fig. 4A,B). The health outcomes of this taxa loss remain to be determined.

Among suckling neonates, TCC exposure led to microbial diversity loss. Within cecum samples collected from exposed neonates, visually the overall alpha diversity declined overtime with significant suppression of phylogenetic diversity at PND 16 (Fig. 4B) compared to controls. A similar pattern developed when neonatal beta diversity was evaluated, whereby in control and TCC exposed neonates, an initial stochastic pattern emerged at PND 3. At PND 6, an overall restructuring occurred where control and TCC exposed communities converged. Starting from PND 12, communities separated based on exposure status and became significantly different at PND 16 (Fig. 5, Supplementary Fig. S2). The overall restructuring that occurred among samples collected from control neonates at PND 6 was interesting, though may provide an indication of the normal colonization process. Using Friend leukemia virus B mice, Pantoja-Feliciano *et al*. demonstrated suppressed diversity at PND 3 and 9, compared to day 1, that increased again to levels similar to dams at PND 21^25^. Additionally, Palmer *et al*. reported that the mean Pearson’s correlation between human infant and adult fecal microbiota increased from day 0 until around day 5 post-birth, when an apparent population rearrangement occurred resulting in the divergence from adult samples^7^. Thereafter, infant microbial profiles again correlated more closely to adults throughout the first 18 months of life.

We demonstrated that distances of samples from control neonates at PND 3 clustered around microbiota of control dams at GD 18 (Fig. 6A). By PND 6, a population shift occurred, with movement of neonates away from that of dams. At PNDs 12 and 16, the neonatal distance from dams decreased at each respective time-point. Microbiota may initially be acquired and only those microbes that can occupy the niche specific to the infant gut will colonize^5^. We postulate that among control neonates, the initial dam-neonate similarity reflects the microbiota transferred either from the dam or environment over the first few days of life. At PND 6, only those microbes that can occupy the neonatal cecum biome propagate. This in turn produces a more hospitable microbial environment, driving increased diversity and similarity to adult dam samples at PNDs 12 and PND 16.

The distance similarity noted between control dams at GD 18 and neonates at PND 3 was not demonstrated among TCC exposed dams and their neonates (Fig. 6B). Here, among exposed neonates, early life TCC exposure constrained the progression to a more diverse state. One limitation of this study design was that samples were not collected prior to PND 3. Thus we cannot comment on the potential similarity of TCC exposed neonatal microbiota to dams at the time of delivery. Furthermore, because exposure during gestation and lactation was not separated, we could not dissect the effect of early microbial restriction at gestation or lactation individually. Previously, we demonstrated that TCC could cross the placental barrier and was detected in the amniotic fluid^21^. Further, research now indicates that microbial establishment may occur prenatally^26^. Thus, we could not rule out the possibility that restriction demonstrated in TCC exposed neonates was an outcome of gestational exposure. Cesarean-section or a cross-fostering design could be utilized in the future to separate the effect of TCC exposure between gestation and lactation. Further, sample collection at the time of parturition would provide further insight into the normal colonization process at early life stages.

Additional limitations such as the small sample size (n = 4 dams, and n = 3 neonates) and the fact that the neonatal samples were pooled is noted. Pooling samples reduces variability, but could also reduce the overall sample number and statistical power. However, because TCC was administered through the chow to dams and neonatal exposure occurred through the milk to the whole litter, statistical analysis based on the whole litter instead of using individual neonates within each litter has been proposed^27^. Specifically, it might not be an ideal situation to collect two sibling neonates from a single litter and consider these two animals as separate experimental units. In this study, we pooled neonates collected from separate cages within each treatment group in order to reduce the variability that might be demonstrated if only a single neonate were collected at each time point. Future investigation with increased dam numbers producing larger neonatal sample size would strengthen the power of these studies.

The infant gut is first colonized by facultative anaerobes such as *Enterobacteriaceae* that lower the redox potential allowing for growth of strictly anaerobic bacteria^28^. Among neonates at PND 3, *Enterobacteriaceae* dominated in both control and treated groups (Fig. 7B, Table 4). After PND 6, *Bacteroidaceae* gained a stronghold in control samples becoming significantly enhanced compared to exposed samples overtime (Table 4). In contrast, *Enterobacteriaceae* maintained dominance in TCC exposed samples from PND 6 until the end of the study period and repeated measures analysis revealed a significant effect of treatment group compared to control samples (Table 4). The relative contribution of *Enterobacteriaceae* may explain the convergence at PND 6. Because TCC shows selective efficacy for Gram-positive bacterial strains^24^, if mostly Gram-negative bacteria dominated at PND 6 in both groups, the effect of TCC may be minimal. However, with consistent exposure few Gram-positive bacteria, for example, may colonize overtime contributing to the diversity difference between the two groups.

Currently the specific cause of diarrhea in exposed animals is elusive. It is possible that exposure to TCC allowed for colonization of pathogenic members of the *Enterobacteriaceae* family and subsequent infection. Because samples were pooled, if only certain neonates of a pool were colonized by a pathogenic strain of *E.coli*, for example, the total contribution might be minimized and infection based solely on microbial abundance may be overlooked. However, all TCC exposed neonates displayed distended abdomens (Fig. 8B) and diarrhea, indicating that if infection/diarrhea was the result of an overgrowth of a certain member of the *Enterobacteriaceae* family, pooling samples would not mask the effect. *Enterobacteriaceae* bloom in the gut microbiota is documented among human infants in response to prescription antibiotics and is associated with potentially life threatening diseases such as necrotizing enterocolitis^29^,^30^,^31^. Furthermore, a reduction in the ratio of *Bacteriodeace* to *Enterobacteriaceae* of the human infant gut is indicated in later-life health outcomes, such as food sensitivities^32^. Collectively, our results should drive future research regarding both short and long-term health consequences related to TCC exposure in humans, specifically during early life.

It was interesting to note that exposed neonates showed distended abdomens (Fig. 8B) with mustard-colored diarrhea, though no apparent histopathological differences were identified (Fig. 8C,D). Currently, the mechanism of TCC-induced diarrhea among the neonates is unknown. However, given its antimicrobial nature, TCC may act similarly to prescription antibiotics. The use of many prescription drugs, including antibiotics, can lead to diarrhea onset, commonly without organic lesions^33^. Antibiotic-associated diarrhea (AAD) is unexplained diarrhea that is associated with antibiotic administration^34^. The mechanisms of AAD are diverse and may be related to the pharmacokinetic properties of the drug itself or to suppression of the gut microbiota. Future investigation may benefit from the use of gnobiotic animals to rule out the interaction of TCC on the gut microbiota to determine whether diarrhea was a primary effect of TCC exposure or secondary to its action on the gut microbiota.

## Conclusion

Oscillations in the gut microbial community structure can occur with exposure to prescription antibiotics leading to dysbiosis of the gut ecosystem^35^. These compositional changes can induce opportunistic pathogen overgrowth resulting in infectious disease (i.e. *C. difficile* infection) in the short-term and chronic disease (i.e. asthma) throughout life. Like prescription antibiotics, we demonstrated the ability of a non-prescription antimicrobial TCC, to induced gut microbial dysbiosis during sensitive exposure windows in a rat model. Collectively, our results add to the growing public concern related to the potential human health impact of non-prescription antimicrobial exposure and should guide regulatory agencies in policy decisions regarding the use of non-prescription antimicrobials in personal care products during critical physiological stages.

## Materials and Methods

### Animals and Husbandry

Timed-pregnant Sprague Dawley (SD) rats were purchased from Harlan Laboratory (Dublin, VA). The day after mating was designated as gestational day (GD) 1. Upon arrival, animals were weight ranked and randomly assigned to control or treatment groups (n = 4/group). Animals were housed individually under specified conditions (12:12-hour light cycle, temperature of 20 °C to 22 °C, and relative humidity of 40% to 50%) with ad libitum access to water and commercial Harlan ground 2020X chow or 2020X supplemented with 0.1% w/w TCC (purity 99%, Sigma Aldrich, St Louis, Missouri) daily from GD 4 until 16 days after delivery. This period was chosen as we demonstrated that TCC could cross the placental barrier and accumulate in the milk of lactating rats^21^. We previously demonstrated that the serum TCC concentration of pregnant rats after oral exposure to 0.2% w/w TCC was similar to or within an order of magnitude of the concentrations reported in the blood of human volunteers, specifically in those who were regular users of TCC containing soap^21^,^36^. Parturition occurred naturally. The Animal Use and Care Committee at the University of Tennessee, Knoxville, approved all study protocols. All methods were conducted in accordance with the Institutional Animal Care and Use Committee (IACUC) guidelines. This investigation was conducted in an animal facility fully accredited by the Association for Assessment and Accreditation of Laboratory Animal Care.

### Fecal/cecum Sample Collection

Fecal samples from dams (n = 4/group) and cecum content (n = 3/group) from neonates were collected at designated dates throughout gestation and lactation. Briefly, for fecal collection, an individual dam was removed from the home cage and placed in a clean cage free of bedding. The tail of the rat was lifted to facilitate the discharge of feces. Stainless steel forceps were used to collect fecal pellets immediately after the samples were produced. All tools were autoclaved prior to use and changed between cages. Fecal samples were collected at baseline (GD 4), 7 days post-treatment (GD 11), 14 days post-treatment (GD 18), and 16 days after delivery (AD) corresponding to 34 days after the initiation of exposure regimen. No fecal samples were collected from dams between GD 18 and any days prior to the final day of the study to reduce disturbance prior to the delivery and during lactation.

Collection of cecum content from neonates was a terminal procedure. Samples were collected between 8:00 AM and 12:00 PM on the day of sacrifice. At postnatal day (PNDs) 3, 6, 12 and 16, within each group, two female neonates were randomly selected from each dam. Cecum content from each neonate was removed and combined into three pools so that no individual pool contained two neonates born to the same dam. The maternal origin of the composition of each pool was made consistent at each subsequent collection date. In other words, if cecum content from a neonate born to a specific dam was added to a designated pool on PND 3, cecum content from an additional neonate born to the same dam was used to create the same pool on PND 6. Fecal/cecum samples were snap frozen immediately following collection and stored at −80 ° C until analysis.

### Neonatal Histology

At PND 12, neonatal gastrointestinal tracts (jejunum and cecum) were collected from male neonates with or without TCC exposure during lactation and fixed in 10% formalin. Tissue sections were examined with hematoxylin-eosin (H&E) staining. A board-certified pathologist, blinded to treatment status, evaluated histological changes.

### DNA Isolation, Amplification, and 16S rRNA Sequencing

#### DNA Extraction, Amplification and Clean-up

DNA was extracted from frozen fecal/cecum samples with the Power fecal DNA isolation kit (Mo Bio Laboratories, Inc. Carlsbad, CA) following manufacturer’s instructions. Extracted DNA samples were quantified with Nanodrop 1000™ and stored at −80 °C until PCR amplification. DNA was amplified by targeting the V4 region of the bacterial 16S rRNA gene as described by Caporaso *et al*.^37^.

The initial PCR product was purified with DNA gel electrophoresis to remove DNA impurities and primer dimers. The concentration of purified amplicon product was measured with Qubit dsDNA HS Assay Kit (Life Technologies, Carlsbad, CA) and normalized to an equal concentration to create a single amplicon pool.

### Bacterial Barcoded Amplicon Library Preparation, Sequencing and Sequence Analysis

#### Beads Clean-up

Pooled amplicons were purified with SPRIselect (Beckman Coulter, Inc., Indianapolis, IN) following the manufacturer’s protocol (Next-flex™ 16S V4 Amplicon Seq-kit manual). The products were analyzed with Agilent High Sensitivity DNA Analysis (CHIP) Kit for quality assurance on a 2100 Bioanalyzer (Agilent, Santa Clara, CA).

##### Library Quantification and Illumina Sequencing

The pooled amplicon library concentration was quantified with the Illumina Library Quantification kit (KAPA Biosystems, Boston, MA) prior to sequencing. Quantitative

PCR was performed with the KAPA SYBR^®^ FAST qPCR Master Mix (2X). The amplicon library was diluted to a starting concentration of 10 nM and sequenced on the Illumina MiSeq sequencer (Illumina, Inc., San Diego, CA).

#### Sequence Data Analysis

The resulting raw sequencing data was analyzed using the QIIME (v1.8.0) pipeline^38^. Unless otherwise stated all python scripts reside within QIIME. The script, join_paired_ends.py, was used to generate the assembled paired-end reads. Next, paired-end sequences were demultiplexed and quality filtered with Phred score no less than 20. The UCHIME program was used to detect chimeras on assembled reads via identify_chimeric_seqs.py^39^. Operational taxonomic units (OTU) were generated using the script, pick_open_reference_otus.py, with 97% similarity via USEARCH^40^,^41^. The OTU taxonomy was assigned using the Ribosomal Database Project (RDP) classifier^42^ with the May 2013 Greengenes release in QIIME^43^,^44^, and then aligned via PyNAST^45^. Any OTU present at less than 0.005% of the total read count was filtered to remove the potential influence of spurious OTUs^46^,^47^. The resulting filtered output was used to make a phylogeny (make_phylogeny.py). The phylogeny was then rooted to Bacteroidetes. All samples were rarefied at a minimum sequencing depth of 55,000 OTUs. The script alpha_rarefaction.py was used to confirm the appropriate minimum sequencing depth across samples. 16S datasets were deposited in the Sequence Read Archive under accession number: SRP067613.

### Statistics

Statistical analysis was conducted in R (version 3.1.2) using Phyloseq unless otherwise noted^48^. Microbial community composition by treatment group at each fecal/cecum collection date was visualized using Principal Coordinate Analysis (PcoA) and boxplots constructed with weighted UniFrac distances^49^. Community level statistical significance was tested with the nonparametric Adonis function in the Vegan package at each individual time-point^50^,^51^. Adonis permutations were stratified by collection date among neonates in the Vegan package to account for sampling across time. A repeated measures permanova was conducted on dam samples stratified by rat ID using the BiodiversityR package, with separate whole and sub-plot analyses^52^. Post-hoc analysis of repeated measures Adonis results were analyzed with the Vegan package to dissect significant time-treatment interactions. Within sample richness and evenness were estimated using Shannon’s index. Faith’s phylodiversity (PD) metric was calculated via QIIME^53^. Dam alpha diversity estimates were analyzed using two-way ANOVA with repeated measures and neonate alpha diversity was analyze by ANCOVA using dam GD 18 alpha diversity as a covariate in SigmaPlot (version 13) with Bonferroni post-hoc test. Data were presented as group mean ± SEM. Relative abundance of OTUs at the phyla and family level were visualized with Phyloseq^48^. Statistical significance was set at alpha = 0.05. Relative abundance was analyzed statistically using the glm function in R. Time and treatment fixed effects were analyzed with ANOVA and Fisher’s LSD mean separation (lsmeans package)^54^.

## Additional Information

**How to cite this article**: Kennedy, R. C. *et al*. Temporal Development of Gut Microbiota in Triclocarban Exposed Pregnant and Neonatal Rats. *Sci. Rep.* **6**, 33430; doi: 10.1038/srep33430 (2016).

## Supplementary Material

Supplementary Information

## Figures and Tables

**Table 1:** Effect of TCC on Relative Abundance of Dam Gut Microbial Phyla.

Phylum*	Stain	Group	Least Squares Means**			
			GD 4	GD 11	GD 18	AD 16
Bacteroidetes^C^	Gram −	Control	42.6	24.6	30.7	46.4
		Exposed	41.1	39.3	65.2	20.6
Firmicutes^C^	Gram +	Control	48.0	71.9	65.5	48.7
		Exposed	56.0	56.4	30.1	69.5
Proteobacteria^ABC^	Gram −	Control	2.7	<1	2.0	2.1
		Exposed	1.3	4.1	4.3	9.3
Tenericutes^AB^	Gram −	Control	2.5	1.4	<1	1.7
		Exposed	1.1	<1	<1	<1

A = Effect of time, p < 0.05; B = Effect of treatment, p < 0.05; C = Time-treatment interaction, p < 0.05; ^*^Data displayed comprise ≥ 1% of the relative abundance.

^**^Least squares means displayed as percentage; GD = gestational day; AD = after delivery.

**Table 2:** Effect of TCC on Relative Abundance of Dam Gut Microbial Families.

Family*	Stain	Least Squares Means**				
		Group	GD4	GD 11	GD 18	AD 16
[Paraprevotellaceae]^ABC^	Gram −	Control	<1	<1	<1	<1
		Exposed	<1	1.5	4.5	<1
Alcaligenaceae^C^	Gram −	Control	2.1	<1	1.3	<1
		Exposed	<1	2.7	2.0	<1
Bacteroidaceae^ABC^	Gram −	Control	2.5	3.8	7.7	7.3
		Exposed	1.1	34.8	56.1	18.9
Clostridiaceae^B^	Gram +	Control	2.0	3.8	3.4	2.4
		Exposed	1.2	<1	<1	<1
Desulfovibrionaceae^ABC^	Gram −	Control	<1	<1	<1	<1
		Exposed	<1	<1	2.0	2.5
Enterobacteriaceae^ABC^	Gram −	Control	<1	<1	<1	<1
		Exposed	<1	<1	<1	6.0
Lactobacillaceae^B^	Gram +	Control	1.5	3.2	1.6	4.4
		Exposed	2.2	<1	<1	1.7
Porphyromonadaceae^ABC^	Gram −	Control	1.5	<1	<1	<1
		Exposed	<1	2.8	4.2	1.3
Rikenellaceae^BC^	Gram −	Control	<1	2.8	1.9	4.3
		Exposed	<1	<1	<1	<1
Ruminococcaceae^AC^	Gram +	Control	18.9	17.8	19.6	15.8
		Exposed	16.8	33.8	10.1	21.4
S24−7^ABC^	Uncultured	Control	37.4	14.9	18.5	32.5
		Exposed	38.5	<1	<1	<1
Turicibacteraceae^AB^	Gram +	Control	1.1	1.0	1.1	<1
		Exposed	1.1	<1	<1	<1

A = Effect of time, p < 0.05; B = Effect of treatment, p < 0.05; C = Time-treatment interaction, p < 0.05; ^*^Data displayed comprise ≥ 1% of the relative abundance; ^**^Least squares means displayed as percentage; GD = gestational day; AD = after delivery.

**Table 3:** Effect of TCC on Relative Abundance of Neonate Gut Microbial Phyla.

Phylum*	Stain	Group	Least Squares Means**			
			PND 3	PND 6	PND 12	PND 16
Bacteroidetes^AB^	Gram −	Control	22.6	<1	50.3	64.8
		Exposed	9.0	<1	9.4	21.3
Firmicutes^A^	Gram +	Control	41.0	11.9	7.2	25.2
		Exposed	37.7	5.8	2.4	<1
Proteobacteria^B^	Gram −	Control	35.3	87.3	34.0	7.5
		Exposed	52.7	94.0	88.2	78.0

A = Effect of time, p < 0.05; B = Effect of treatment, p < 0.05; C = Time-treatment interaction, p < 0.05; ^*^Data displayed comprise ≥ 1% of the relative abundance.

^**^Least squares means displayed as percentage; PND = postnatal day.

**Table 4:** Effect of TCC on Relative Abundance of Neonate Gut Microbial Families.

Family*	Stain	Group	Least Squares Means**			
			PND 3	PND 6	PND 12	PND 16
Bacteroidaceae^ABC^	Gram −	Control	12.8	<1	48.4	39.6
		Exposed	7.5	<1	1.0	18.1
Enterobacteriaceae^B^	Gram −	Control	33.3	82.1	32.4	7.2
		Exposed	51.3	93.7	88.0	77.9
Pasteurellaceae^ABC^	Gram −	Control	<1	5.2	1.5	<1
		Exposed	<1	<1	<1	<1
S24−7^ABC^	Uncultured	Control	6.9	<1	1.9	16.5
		Exposed	<1	<1	<1	<1

A = Effect of time, p < 0.05; B = Effect of treatment, p < 0.05; C = Time-treatment interaction, p < 0.05; ^*^Data displayed comprise ≥1% of the relative abundance.

^**^Least squares means displayed as percentage; PND = postnatal day.
