# Supplementary material for: Temporal Development of Gut Microbiota in Triclocarban Exposed Pregnant and Neonatal Rats
Source: Sci Rep. 2016 Sep 20;6:33430. doi: 10.1038/srep33430 (PMC5028839; doi:10.1038/srep33430)
Supplement: Supplementary Information [file srep33430-s1.pdf]

Temporal Development of Gut Microbiota in Triclocarban Exposed Pregnant and Neonatal Rats

Rebekah C. Kennedy[1,2]*, Russell R. Fling[3]*, Michael S. Robeson[4,5], Arnold M. Saxton[6], Robert L. Donnell[7], John L. Darcy[8], David A. Bemis[7], Jiang Liu[3], Ling Zhao[9] and Jiangang Chen[1]**

[1]Department of Public Health, [2]Comparative and Experimental Medicine, [3]Department of Microbiology, The University of Tennessee, Knoxville, TN 37996, USA, [4]Department of Fish, Wildlife & Conservation Biology, Colorado State University, Fort Collins, CO 80523, USA, [5]USDA APHIS, National Wildlife Research Center, Fort Collins, CO 80521, USA, [6]Department of Animal Science, [7]Department of Biomedical and Diagnostic Sciences, The University of Tennessee, Knoxville, TN 37996,USA, [8]Department of Ecology and Evolutionary Biology, University of Colorado, Boulder, CO 80309, USA, [9]Department of Nutrition, The University of Tennessee Knoxville, TN 37996, USA.

*Two authors contributed equally, **corresponding author

Supplementary Figures

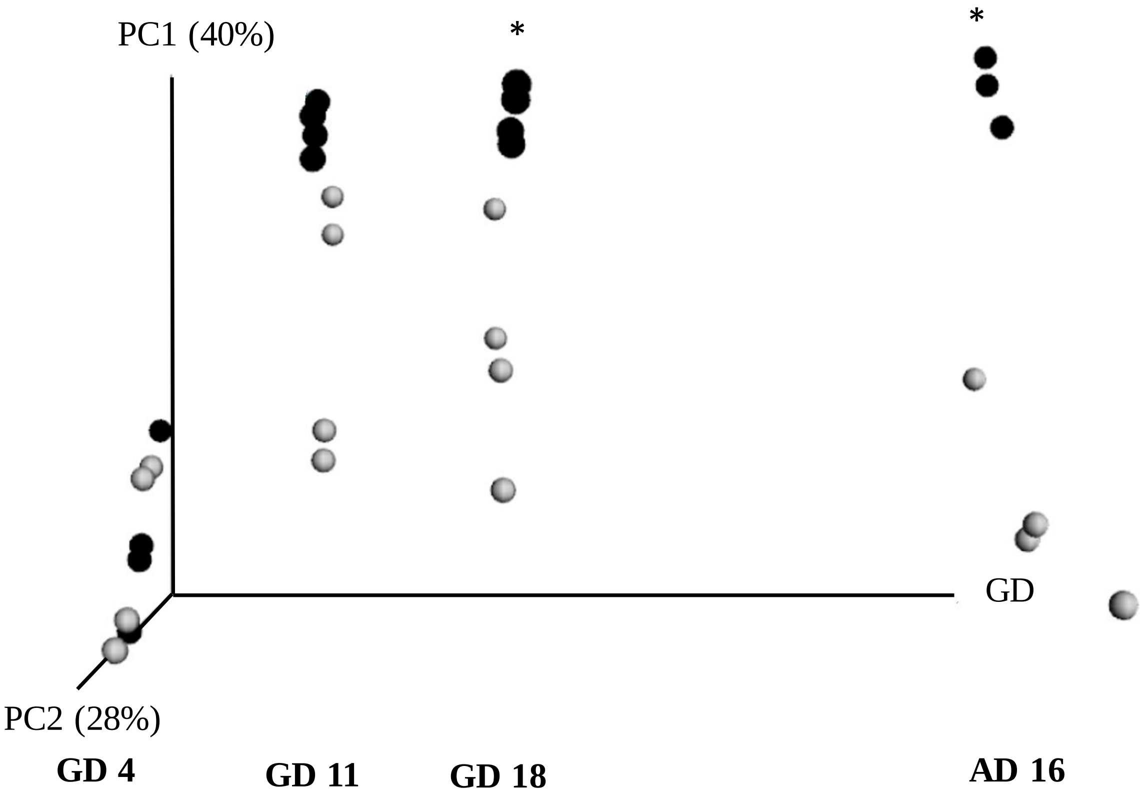

Supplementary Figure 1. Beta diversity of dams during gestation and lactation. Principal coordinate analysis of weighted UniFrac distances is shown among dams at GDs 4 (baseline), 11, 18 and 16 days after delivery (AD) (control: gray circle, 0.1% w/w TCC: black circle; n=4/group). Statistical significance of community level microbial distance was analyzed with ADONIS, in the Vegan package, at each collection date [50,51]. Repeated measures analysis was conducted and significant time-treatment interactions were investigated with the Vegan package. (*) indicates statistical significance at each time point relative to controls. Samples are fixed by gestational day (GD)/ collection date after delivery (AD) on the third axis**.**

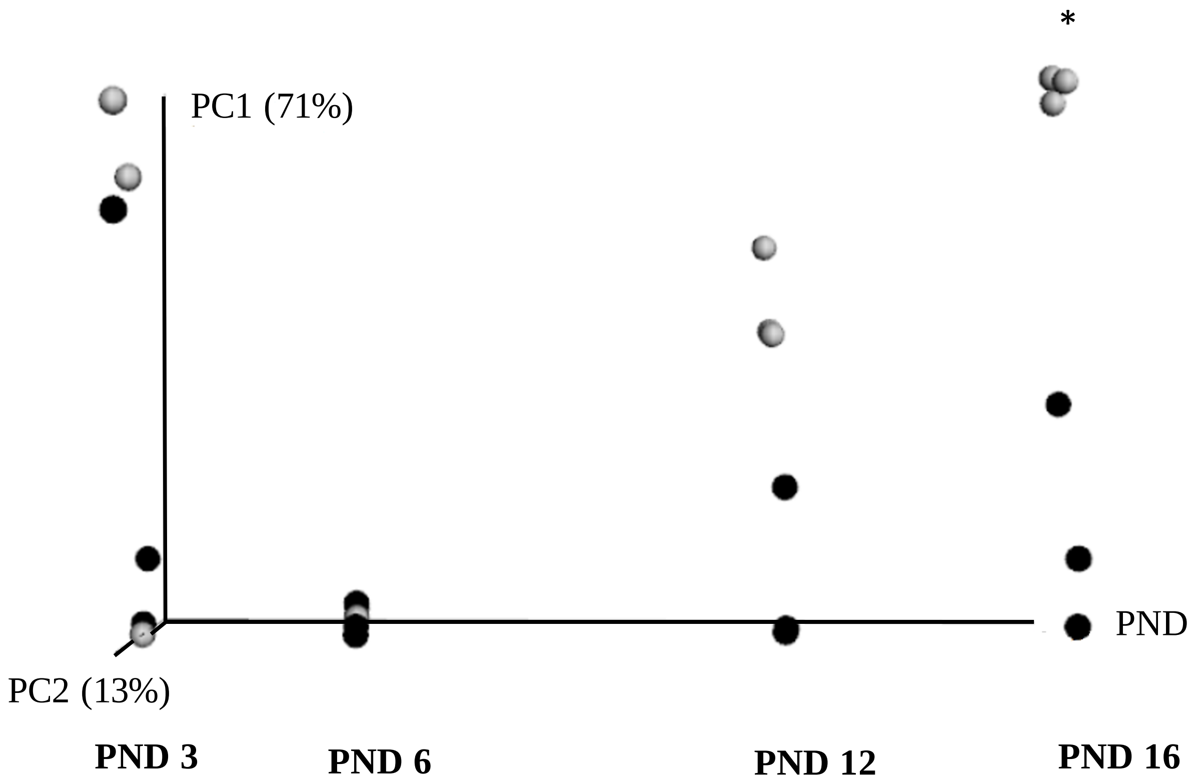

Supplementary Figure 2. Beta diversity of neonates during lactation period. Principal coordinate analysis of weighted UniFrac distances is shown at PNDs 3, 6, 12 and 16 (control: gray circle, 0.1% w/w TCC: black circle; n= 3/group). Community level statistical significance was analyzed using ADONIS, in the Vegan package[50,51]. Repeated measures analysis was conducted and significant time-treatment interactions were investigated with the Vegan package. (*) indicates statistical significance at each time point relative to controls. Samples are fixed by postnatal day (PND) on the third axis.
